# Esophageal Impedance-pH Monitoring and Pharyngeal pH Monitoring in the Diagnosis of Extraesophageal Reflux in Children

**DOI:** 10.1155/2019/6271910

**Published:** 2019-03-03

**Authors:** Anna Plocek, Beata Gębora-Kowalska, Jakub Białek, Wojciech Fendler, Ewa Toporowska-Kowalska

**Affiliations:** ^1^Department of Pediatric Allergy, Gastroenterology and Nutrition, Medical University of Lodz, Lodz, Poland; ^2^Department of Pediatric Emergency Medicine, Medical University of Lodz, Lodz, Poland; ^3^Department of Biostatistics and Translational Medicine, Medical University of Lodz, Lodz, Poland; ^4^Department of Radiation Oncology, Dana-Farber Cancer Institute, Boston, MA, USA

## Abstract

Various clinical symptoms are attributed to extraesophageal reflux disease (EERD). Multichannel intraluminal impedance-pH monitoring (MII-pH) is considered to correlate symptoms with acid and nonacid gastroesophageal reflux (GER) events. Pharyngeal pH monitoring (Dx-pH) is considered to correlate the decrease in the pH level in the oropharynx with reported symptoms and to diagnose supraesophageal reflux. We aimed to assess the correlation between acid reflux episodes recorded by Dx-pH and GER detected via MII-pH in children with suspected EERD. The study enrolled 23 consecutive children (15 boys and 8 girls; median age 8.25 [range 3-16.5] years) with suspected EERD. MII-pH and Dx-pH were conducted concurrently in all patients. A total of 1228 reflux episodes were recorded by MII-pH. With the antimonic sensor placed inside the impedance probe, 1272 pH-only reflux episodes were recorded. Of these, 977 (76.81%) were associated with a retrograde bolus transit. Regarding GER, 630 full-column episodes extended to the most proximal pair of impedance sensors; 500 (83.33%) demonstrated an acidic character. The following acid reflux numbers were determined by the Dx-pH system: for pH < 4, *n* = 126; pH < 4.5, *n* = 136; pH < 5, *n* = 167; and pH < 5.5, *n* = 304, and for a decrease in pH > 10% relative to the baseline, *n* = 324. There was no significant correlation between the number of pharyngeal reflux episodes detected by Dx-pH and that of GERs identified by MII-pH. The proportion of oropharyngeal pH events that were temporally related to a GER episode increased with the extended pH criteria. The highest proportion was observed for a pH decrease of ≥10% from the baseline and did not exceed 5.2%. The application of the extended pH criteria in the Dx-pH system resulted in an increase in the number of diagnosed laryngopharyngeal refluxes; most were not temporally associated with GER episodes confirmed by MII-pH. Thus, the efficacy of the exclusive application of Dx-pH for supraesophageal gastric reflux diagnosis is uncertain.

## 1. Introduction

Gastroesophageal reflux (GER) disease (GERD) in children occurs in two forms: esophageal and extraesophageal [1]. Various clinical symptoms are attributed to extraesophageal reflux disease, which involves the regurgitation of gastric contents above the upper esophageal sphincter (UES). These symptoms may include hoarseness, chronic cough, postnasal drip syndrome, or globus pharyngeus, as well as defined syndromes such as asthma, sinusitis, otitis media, and laryngitis. However, the only established associated symptoms in children include Sandifer's syndrome and dental erosion, while bronchopulmonary, laryngotracheal, pharyngeal, otic, and nasal syndromes as well as acute conditions in infants such as apnea, bradycardia, and apparent life-threatening event (ALTE; currently, the American Academy of Pediatrics recommends the replacement of the term ALTE with a new term, brief resolved unexplained event—BRUE [2]) are treated as potential but poorly documented associations. It is challenging to derive a reliable diagnosis of extraesophageal reflux disease as it requires complex differential diagnostic steps and the deployment of research methods that facilitate the association of disease symptoms/syndromes with the phenomenon of GER in temporal terms.

In the literature, extraesophageal reflux disease is also referred to as atypical reflux disease, supraesophageal reflux disease, laryngeal reflux, pharyngoesophageal reflux, or, pursuant to the definition by AAOHNS (American Academy of Otolaryngology-Head and Neck Surgery), laryngopharyngeal reflux (LPR) [3]. The notion of supraesophageal gastric reflux (SEGR) was proposed by Chiou et al. [4], while the proposed definition—a decrease in pH < 4.0 in the oropharynx with a preceding or concurrent episode of distal reflux—pertained to multichannel intraluminal impedance-pH monitoring (MII-pH), which allows the detection of GER regardless of the pH level. According to a previous protocol provided by the European Pediatric Impedance Working Group (EURO-PIG) [5] and the current joint guidelines of the North American Society for Pediatric Gastroenterology, Hepatology, and Nutrition (NASPGHAN) and the European Society of Pediatric Gastroenterology, Hepatology, and Nutrition (ESPGHAN) [6], MII-pH is considered to correlate troublesome symptoms with acid and nonacid GER events. Since 2009, pharyngeal reflux has been diagnosed with the so-called pharyngeal pH monitoring (the Dx-pH Measurement System), which is a system for recording the pH of liquid or aerosolized droplets in the posterior oropharynx, facilitating the correlation of a decrease in the pH level with symptoms signaled by the patients [7]. The utility of Dx-pH in the diagnosis of extraesophageal reflux disease is controversial, with some researchers questioning the viability of GER diagnoses based on this method [8].

The objective of our study was to assess the correlation between episodes of acid reflux recorded via Dx-pH and MII-pH in children with suspected extraesophageal reflux disease.

## 2. Materials and Methods

The study was conducted in accordance with the Helsinki Declaration and approved by the Ethical Committee of the Medical University of Lodz. Parents and children aged above 16 years provided written informed consent prior to participation in the study. The study comprised 23 consecutive children (15 boys and 8 girls) aged 3-16.5 years (median age 8.25 years) who were admitted to the Department of Pediatric Allergology, Gastroenterology, and Nutrition, Medical University of Lodz, between January and December 2016, with suspected extraesophageal reflux disease.

The inclusion criteria were chronic disease, either lasting over 2 months or occurring recurrently, extraesophageal symptoms, or disease conditions that could have evolved because of reflux. The exclusion criteria comprised antisecretory therapy with proton pump inhibitors (PPIs) in the last 7 days, congenital gastric/esophageal defects, and surgeries within the area of the esophagus and stomach.

MII-pH (Sandhill Scientific, Highlands Ranch, CO) and Dx-pH (Restech Corp., San Diego, CA) were conducted concurrently in all patients. The MII-pH recording was performed with a portable data logger and a combined impedance-pH catheter. Two types of catheters were deployed: (1) pediatric catheters with a diameter of 2.13 mm/6.4 Fr with 6 impedance channels and 1 pH sensor positioned in the center of the most distal impedance channel (for patients with a height of <150 cm) and (2) catheters for adults with a diameter of 2.3 mm/6.9 Fr with 6 impedance channels and 1 pH sensor positioned in the center of the second most distal impedance channel (for children with a height of >150 cm).

Both disposable and reusable catheters were used. Prior to each examination, reusable catheters were subjected to high-level disinfection, which eliminates vegetative forms of bacteria, viruses, and fungi.

The catheters were precalibrated in buffers with pH of 4 and 7 and inserted via the anterior nares, and the correctness of the catheter placement was confirmed by radiography. Proper localization was required for the pH monitoring sensor to be positioned 3 cm (children < 150 cm) or 5 cm (children > 150 cm) above the proximal edge of the lower esophageal sphincter (LES). The six impedance and pH signals were recorded at 50 Hz (every 0.02 seconds). The patients had fasted for at least 4 hours prior to the examination and did not receive prokinetic drugs or drugs that decreased gastric secretion. PPIs, histamine H_2_-receptor blockers, and prokinetic drugs were stopped 7, 3, and 2 days, respectively, prior to the test in accordance with current guidelines [9].

Upon positioning the MII-pH catheter inside the esophagus, a Dx-pH probe was inserted through the same nares and positioned at the posterior wall of the pharynx at the level of the uvula. The Dx-pH probe is fitted with an inbuilt flashing LED that allows it to be located without radiography. pH was measured in real time every 0.5 seconds in both liquid and gaseous environments using a sensor at the distal tip of the probe. The data collected by the probe were transmitted to the logger by radio waves, allowing the patient to move freely within a radius of 10 meters from the device.

Prior to each monitoring, internal clocks of both data loggers were synchronized, allowing a simultaneous recording of MII-pH and Dx-pH. The patients were instructed to pursue standard daily activities during the monitoring, to abide by their usual sleep regime, and to keep to their daily dietary routines (barring acidic dishes/beverages). Throughout the 24-hour-long recording, the patients or their caregivers registered data concerning symptoms experienced, ingested meals, and body positions using icons placed on the logger (according to the pattern attached to each device). In addition, the data were noted in the patient's paper daily log. After 24 hours, the catheters were removed, and the data obtained were downloaded from the logger to the computer for test result development. The MII-pH recordings were analyzed using a commercial software program (BioView Analysis, Sandhill Scientific, Highlands Ranch, CO), while oropharyngeal pH recordings were examined with another software program (DataView Lite, Respiratory Technology Corp., San Diego, CA). Upon each automated analysis, all recordings were reassessed manually. Meal periods were excluded from the scope of the analysis for both tests.

It was determined that a liquid reflux episode could be diagnosed based on MII-pH once a retrograde decrease in intraesophageal impedance of ≥50% of the baseline in ≥2 distal impedance channels occurred (impedance-detectable event). An acid reflux episode was defined as a decrease in the pH level ranging from an initial value of >4.0 to a value of <4.0 upon the physical presence of refluxate (as confirmed by impedance sensors). GER incidents for which pH value ranged from 4–7 were classified as weakly acidic refluxes, while those equal to or exceeding pH ≥ 7.0 were classified as weakly alkaline. A pH-only reflux episode was defined as a fall in distal pH to <4.0 lasting at least 5 seconds detected by the pH sensor, in the absence of reflux detected by impedance monitoring (GER in pH monitoring). A reflux incident was determined to be proximal if detected by impedance sensors positioned at least 15 or 11 cm above LES for adult and pediatric catheters, respectively (full-column reflux).

The Dx-pH system software calculates the percentage of time when the pH level in the pharynx drops below the adopted cutoff threshold, the number of such episodes, and their duration expressed in minutes. The above parameters allow the calculation of the Ryan score which is the ultimate index of LPR incidence [10].

In conventional terms, LPR episodes are defined as a decrease in pH to <4. Alternatively, they are related to a decrease in the pH level below 5.5, 5.0, and 4.5 or a pH drop of ≥10% from the baseline.

In the following stage, a temporal association between a decrease in the pH level in the oropharynx recorded by Dx-pH and the esophageal reflux episodes recorded by pH-impedance (events of distal GER or proximal GER defined as full-column acid) was evaluated. It was assumed that the pH level decreases detected by the Dx-pH system were associated with GER if they occurred within a maximum of 2 minutes from the onset of the MII-pH reflux episode.

The results were subjected to statistical analysis. Continuous variables are presented as means ± standard deviations or medians depending on the normality of the distribution. The correlation between MII-pH and Dx-pH findings was evaluated for various pH cutoff points via Spearman's rank correlation test. The positive predictive value (PPV) and sensitivity of the Dx-pH system were evaluated with regard to reflux incidents detected using the MII-pH method. A *p* value of <0.05 was considered statistically significant. 95% confidence intervals (95% CI) were calculated where possible.

## 3. Results

The children manifested extraesophageal symptoms such as cough (*n* = 12), throat clearing (*n* = 8), burning sensation in the pharynx (*n* = 3), sore throat (*n* = 2), hoarseness (*n* = 2), choking (*n* = 1), recurrent laryngitis (*n* = 1), and globus sensation in the pharynx (*n* = 1). Nineteen (82.61%) patients had ailments of the gastrointestinal tract such as heartburn, abdominal pain, belching, and vomiting. A typical reflux syndrome [11] was detected in 4 (17.39%) children.

The mean duration of the MII-pH and Dx-pH recordings was 21.14 ± 1.39 hours. A total of 1228 reflux episodes were recorded with MII-pH. With the antimonic sensor (classic pH monitoring) placed inside the impedance probe, 1272 pH-only reflux episodes were recorded. Of these, 977 (76.81%) were associated with a retrograde bolus transit (detected via impedance). There were 630 GER episodes which extended to the most proximal pair of impedance sensors and were determined to be full-column; 500 (83.33%) of which manifested an acidic character.

The following acid reflux numbers were determined with the Dx-pH system, depending on the cutoff point: for pH < 4, *n* = 126; for pH < 4.5, *n* = 136; for pH < 5, *n* = 167; and for pH < 5.5, *n* = 304, and for a decrease of pH ≥ 10% relative to the baseline, *n* = 324. The highest percentage of pharyngeal refluxes detected by Dx-pH, which also involved swallowing recorded by MII-pH, was recorded for reflux episodes construed as a decrease in pharyngeal pH ≥ 10% relative to the baseline (35.49%). Two hundred and nine (64.51%) of the “pharyngeal refluxes” recorded by Dx-pH were unidentified in the pH-impedance recording (impedance change/pH change).

No significant correlation between pH-impedance and Dx-pH results was observed, irrespective of the assumed pH cutoff points in the interpretation of the Dx-pH recording and the types of GERs recorded by the pH-impedance system. The highest correlation coefficient (in the absence of statistical significance) pertained to the decrease in the pH level in the oropharynx by 10% relative to the baseline value (Table 1).

Owing to the absence of a correlation between the number of LPR and GER episodes assessed in terms of Dx-pH and MII-pH, respectively, the proportion of reflux episodes detected by the Dx-pH system, which were concurrently identified by the impedance and pH sensor of the MII-pH system (Table 2), was analyzed. The proportion of oropharyngeal pH events temporally related to an episode of GER increased with the extended pH criteria. The highest proportion was observed for a pH decrease of ≥10% from the baseline and did not exceed 5.2%.

The highest PPV for the Dx-pH system (43.17%; sensitivity 10.13%) was observed for LPR episodes designated as a decrease in pH > 10% relative to the baseline value (Table 3).

## 4. Discussion

Although the etiopathogenesis of the extraesophageal symptoms of reflux disease is complex and unclear, three major mechanisms have been suggested and include microaspiration (direct), neural reflexes associated with excessive vagus nerve activation (indirect), and vagus nerve-independent axonal stimulation induced by acid gastric contents in the esophagus (inflammation theory).

The role of UES failure in the pathogenesis of supraesophageal reflux is a current topic of discussion. Under physiological circumstances, the UES acts as a natural defense of airways against reflux contents. The UES contracts in reaction to a minor refluxate volume in the esophagus, whereas the regurgitation of a larger volume triggers the closure of the vocal cords mediated by the vagus nerve, physiological apnea, and the relaxation of UES. The refluxate then passes into the pharynx, and the swallowing reflex is initiated, leading to the clearance of the esophagus and the restoration of breathing. This is how the microaspiration of gastric contents containing hydrochloric acid and enzymes (including pepsin) into the airways occurs [12, 13]. An experimental model demonstrated that the direct exposure of the mucosal membrane to hydrochloric acid both in the trachea and esophagus leads to increased pulmonary resistance. This effect proved to be much more potent with regard to the epithelium of the airways: a 5-fold increase in pulmonary resistance was observed in 100% of animals with a hydrochloric acid volume of 1.5 *μ*l in the trachea compared with a 1.5-fold enlargement in 60% of the specimens in response to a volume of 10 ml in the esophagus [14].

Other proposals include the inflammation hypothesis. The excessive exposure of the mucosal membrane to reflux contents may lead to its inflammation and the subsequent denudation and irritation of the embedded receptors in addition to the activation of the sympathetic system, release of inflammatory reaction mediators, including substance P, and consequently, the hyperactivity and contraction of bronchia and the increase in mucosal secretion [15–17].

Currently, there are no unequivocal clinical, gastroscopic, or laryngoscopic criteria for the diagnosis of supraesophageal reflux.

The detection of supraesophageal reflux based on the Dx-pH system requires the assessment of the pH level in the oropharynx using a single-measurement sensor, inserted under direct vision. A study of healthy adult volunteers, with the deployment of this method, proved the adoption of a limit of pH < 4 to be overly restrictive for the purpose of diagnosing “pharyngeal reflux,” and the introduction of alternative criteria (pH < 4.5, pH < 5, pH < 5.5, and pH < 6) boosted the sensitivity of the study [9, 18, 19]. Considering the potential mechanisms behind alterations in the pH level in the oropharynx, Ayazi et al. defined SEGR as a decrease in pH to <5.5 in the vertical position and <4.5 in the horizontal position [18], whereas Feng et al., following the study of 29 Chinese healthy volunteers, suggested that a pH level of 4.5 should be considered the limit for the diagnosis of reflux, under the proviso that LPR be deemed physiological if pH < 4.5 is recorded for a maximum of 1% of the duration of the recording [20]. However, Weiner et al. proposed the reduction in the pH level in the pharynx by at least 10% relative to the baseline value as a diagnostic criterion [7].

Irrespective of the assumed criteria of pharyngeal reflux identification, the Dx-pH method fails to provide tools for tracing the actual direction of refluxate passage from the stomach to the esophagus and, further, to the laryngopharynx. Moreover, it does not help to prove the dependence between pH level alteration in the pharynx and gastroesopharyngeal reflux. While several studies on the assessment of pharyngeal reflux using the Dx-pH method in patients with symptoms of extraesophageal reflux disease are available, only a few were designed to concurrently monitor pH in the oropharynx (Dx-pH) and esophageal pH-impedance, rendering the remaining studies ambiguous in terms of their outcomes. Moreover, only one study concerned children [4]. Based on pH-impedance and Dx-pH, Chiou et al. defined SEGR as a decrease in proximal pH < 4, in conjunction with a preceding or concurrent distal reflux episode [4].

The cutoff value of pH viable for GERD diagnosis, assumed for the pH monitoring test, derives from the proven dependence between heartburn and esophagus exposure to pH lower than 4 [21]. Although the disclosed criteria are universally accepted for the diagnosis of distal GER, they elicit discussion with respect to supraesophageal reflux events. A report revealed that approximately 30% of acid refluxates show a pH > 4.0 upon reaching the proximal esophageal section [22]. The pH gradient from the distal to proximal esophageal segment and, further, to the rhinopharynx is due to the neutralization of the acidic gastric contents by the ingested saliva and the cleansing of esophageal peristalsis. Other reports suggest that a mildly acidic reflux (pH 4.0–7.0) may also lead to pathology within the airways [23–25]. This means that the assumption of pH 4 as the limit value may cause the omission of the exposure of the pharynx and oral cavity to weakly acidic contents and, consequently, to the underestimation of a clinically significant reflux. Furthermore, it was demonstrated that pepsin, which is a component of the refluxate contents, maintains its activity for pH equivalent to 6, which may imply that the destructive effect of pepsin continues with the exposure of the larynx to refluxate contents despite the neutralization of the acid [26].

In the group of children in our study, 126 LPR episodes were detected by the Dx-pH system using the conventional definition of LPR (decrease in pH < 4). The deployment of the extended criteria caused an increase in the recorded LPR episodes, with the highest total number of pharyngeal reflux episodes detected upon the adoption of a decrease in the pH level of >10% relative to the baseline as the cutoff point.

We failed to observe an interdependence between the number of Dx-pH reflux episodes, regardless of the assumed pH level in the oropharynx, and the total number of GER episodes identified via MII-pH (proximal and distal) and the pH sensor (Table 1) in our study population. In addition, the temporal association between LPR and GER was assessed in terms of both the Dx-pH system and pH-impedance. The percentage of consistently “positive” LPR episodes concurrent with GER did not exceed 5.2% (Table 2). The highest temporal consistency for both reflux types and the highest PPV and sensitivity were noted for LPR refluxes detected upon the decrease of pH > 10% relative to the initial value. Our results agree with those of other studies deploying similar methods in adults [27]. A comparable temporal consistency of LPR with all types of refluxes identified by pH-impedance (proximal and distal GER in MII, GER in pH monitoring) was detected.

In the study by Ummarino et al., the percentage of refluxes detected by pH-impedance, which were concurrently identified by Dx-pH, was even lower (1.3%) than that noted in our study [27]. Mazzoleni et al. demonstrated that 120 out of 2394 “impedance” refluxes in 36 patients were concurrently recorded by the pharyngeal Dx-pH sensor [28]. Likewise, Chiou et al., in a study conducted with children, demonstrated that as few as 1% of GER episodes diagnosed by MII-pH corresponded to the episodes detected in the pharynx [4]. The temporal alignment was higher, although it was still low, in a study by Becker et al. who performed a comparative analysis of MII-pH and Dx-pH outcomes in patients suspected with LPR; 36.8% of the instances of reduction in pH in the laryngopharynx were temporally associated with reflux episodes detected in MII-pH [29]. Thus, it is unclear why the reduction in the pH level identified via pharyngeal Dx-pH monitoring does not correspond temporally with reflux episodes recorded by MII-pH.

Chiou et al., as already mentioned, noted the temporal association between LPR episodes and a pH < 4 in the distal esophagus detected by the pH sensor, while there was no association between LPR episodes and reflux identified by pH-impedance [4]. They suggested that the act of swallowing, involving a short-term relaxation of the LES, may cause a minor amount of acid in the stomach to be regurgitated to the utmost distal section of the esophagus. This amount is sufficient for detection by the pH sensor; however, the criteria for the determination of reflux in impedance were not met. It must also be noted that a full-column acid gaseous or aerosolized reflux may occur, leading to a reduction in pH at the level of the pharyngeal sensor. Another possibility is the ingestion of saliva with an acidic pH, causing a decrease in pH at the level of a pharyngeal or esophageal sensor. The literature also contains descriptions of the phenomenon of “pharyngeal pseudo-reflux,” defined as a decrease of pH to <4 detected by a pharyngeal sensor, with no temporal association with the decrease of pH in the esophagus [30].

Our study is limited by the fact that the detection of full-column refluxes in the pH-impedance recording is not equivalent to the passage of this refluxate beyond the UES, which would unequivocally point to SEGR. Reflux episodes that reach the most proximal impedance sensor pair but occur distally with regard to UES may but do not have to affect pH in the pharynx at the level of the uvula. The study by Chiou et al. showed that as few as 3.3% full-column acid reflux episodes identified by pH-impedance were concurrently diagnosed with the Dx-pH probe [4]. Whereas, in the study by Ummarino et al., no refluxate that reached the proximal impedance sensor pair concurred with a decrease in pH in the oropharynx (pH < 5.5) [27]. A more accurate demonstration of the association between GER detected by pH-impedance and the decrease in pH in the oropharynx would require the application of a so-called “esophageal reflux and LPR catheter,” which is equipped with 6 impedance and 2 pH channels, with the proximal sensor positioned at the level of UES (available only in the adult version) [31]. Another shortcoming of this study is the difference in frequency of sampling in the MII-pH and Dx-pH recordings (Dx-pH probe every 0.5 seconds and MII-pH catheter every 0.02 seconds). It might have affected the correlation between the number of refluxes detected by two different methods and temporal association between them.

## 5. Conclusions

The application of the extended criteria of pH assessment in the Dx-pH system resulted in the increase in the total number of diagnosed LPRs. However, the majority of these were not temporally associated with GER episodes confirmed by MII-pH. Thus, the efficacy of the exclusive application of the Dx-pH method for the purpose of SEGR diagnosis and treatment is doubtful.

## Figures and Tables

**Table 1:** Correlation between the number of pharyngeal reflux episodes relative to the assumed pH level detected by the Dx-pH sensor and the number of GER episodes identified with MII-pH (proximal and distal) and the pH sensor.

Number of episodes detected by Dx-pH and MII-pH	*R*	*p*
LPR pH < 5.5 vs. proximal GER in MII-pH	-0.061485	0.780481
LPR pH < 5.5 vs. distal acid GER in pH monitoring	-0.182251	0.405236
LPR pH < 5.5 vs. distal GER in MII-pH	-0.188251	0.389678
LPR pH < 5 vs. proximal GER in MII-pH	-0.112918	0.607964
LPR pH < 5 vs. distal acid GER in pH monitoring	-0.171029	0.435239
LPR pH < 5 vs. distal GER in MII-pH	-0.198517	0.363855
LPR pH < 4.5 vs. proximal GER in MII-pH	-0.052490	0.811990
LPR pH < 4.5 vs. distal acid GER in pH monitoring	-0.193745	0.375732
LPR pH < 4.5 vs. distal GER in MII-pH	-0.164930	0.452029
LPR pH < 4 vs. proximal GER in MII-pH	-0.124503	0.571390
LPR pH < 4 vs. distal acid GER in pH monitoring	-0.071277	0.746559
LPR pH < 4 vs. distal GER in MII-pH	-0.218874	0.315676
LPR upon decrease of pH ≥ 10% relative to baseline vs. proximal GER in MII-pH	-0.201656	0.356162
LPR upon decrease of pH ≥ 10% relative to baseline vs. distal acid GER in pH monitoring	-0.091367	0.678426
LPR upon decrease of pH ≥ 10% relative to baseline vs. distal GER in MII-pH	-0.301609	0.161926

Dx-pH: pharyngeal pH monitoring; MII-pH: multichannel intraluminal pH-impedance; LPR: laryngopharyngeal reflux; GER: gastroesophageal reflux.

**Table 2:** Temporal association between changes in pH in the posterior oropharynx detected by Dx-pH (LPR) and GER episodes identified with impedance (episodes of distal and proximal reflux defined as full-column) and pH sensor.

		LPR vs. proximal GER in MII-pH			LPR vs. distal GER in MII-pH			LPR vs. GER in pH monitoring		
		+/+	-/+	+/-	+/+	-/+	+/-	+/+	-/+	+/-
		Value (95% CI) (%)								
pH level in Dx-pH	<4	1.14 (0.00-2.72)	10.65 (1.24-20.06)	87.06 (76.35-97.78)	0.82 (0.00-2.00)	8.46 (0.07-16.85)	89.91 (80.69-99.12)	1.10 (0.00-2.26)	5.69 (0.00-13.07)	92.10 (83.14-101.06)
	<4.5	1.69 (0.00-4.12)	10.47 (1.28-19.65)	86.15 (74.87-97.42)	1.28 (0.00-3.28)	8.23 (0.00-16.45)	89.22 (79.49-98.96)	1.43 (0.00-3.08)	5.78 (0.00-12.97)	91.37 (81.97-100.77)
	<5	2.17 (0.00-4.81)	14.13 (4.68-23.58)	81.53 (70.02-93.03)	1.62 (0.00-3.77)	10.68 (2.24-19.11)	86.09 (76.13-96.05)	2.01 (0.10-3.91)	6.47 (0.00-13.96)	89.51 (79.64-99.38)
	<5.5	3.06 (0.40-5.73)	27.29 (14.58-40.00)	66.59 (52.05-81.13)	2.41 (0.03-4.79)	21.27 (9.60-32.95)	73.91 (60.76-87.05)	3.54 (1.50-5.58)	13.37 (4.50-22.23)	79.55 (68.11-90.98)
	Decrease ≥ 10%	4.29 (0.65-7.94)	27.44 (14.88-40.00)	63.97 (50.14-77.80)	2.87 (0.09-5.65)	21.68 (9.51-33.86)	72.58 (59.44-85.72)	5.17 (2.51-7.83)	10.98 (3.00-18.95)	78.68 (67.83-89.54)

LPR: laryngopharyngeal reflux; GER: gastroesophageal reflux; MII-pH: multichannel intraluminal pH-impedance; Dx-pH: pharyngeal pH monitoring; CI: confidence interval; “+”: positive result of the Dx-pH system, impedance, or pH monitoring; “-”: negative result of the Dx-pH system, impedance, or pH monitoring.

**Table 3:** Predictive value and sensitivity of the Dx-pH system versus MII-pH.

	Dx-pH vs. MII-pH (proximal GER)		Dx-pH vs. MII-pH (distal GER)		LPR vs. pH monitoring	
pH	PPV	Sensitivity	PPV	Sensitivity	PPV	Sensitivity
	Value (95% CI) (%)					
<4	8.13 (0.00-18.07)	2.58 (0.00-6.87)	8.13 (0.00-18.07)	1.41 (0.00-3.68)	19.30 (2.93-35.68)	2.50 (0.00-5.51)
<4.5	13.36 (0.30-26.41)	4.13 (0.00-11.20)	13.54 (0.40-26.68)	2.37 (0.00-6.46)	24.28 (6.51-42.05)	3.39 (0.00-7.98)
<5	11.88 (1.24-22.52)	5.21 (0.00-13.43)	12.00 (1.29-22.71)	2.97 (0.00-7.59)	33.49 (14.79-52.19)	4.53 (0.00-10.25)
<5.5	13.29 (3.07-23.51)	7.28 (0.00-16.16)	13.89 (3.46-24.32)	4.31 (0.00-9.72)	31.85 (14.96-48.74)	6.86 (0.59-13.14)
Decrease ≥ 10%	21.55 (10.21-32.90)	10.13 (0.00-23.54)	22.09 (10.54-33.65)	5.78 (0.00-13.20)	43.17 (28.13-58.21)	9.66 (0.10-19.23)

Dx-pH: pharyngeal pH monitoring; MII-pH: multichannel intraluminal pH-impedance; LPR: laryngopharyngeal reflux; GER: gastroesophageal reflux; PPV: positive predictive value.

## Data Availability

The data used to support the findings of this study are available from the corresponding author upon request.
